# Development and Validation of the Affective Polarization Scale

**DOI:** 10.5334/irsp.926

**Published:** 2024-06-05

**Authors:** Brandon McMurtrie, Michael Philipp, Ross Hebden, Matt Williams

**Affiliations:** 1 Massey University, NZ; 2 University of Canterbury, NZ

**Keywords:** affective polarization, prejudice, intellectual humility, authoritarianism, negative partisanship

## Abstract

Affective polarization – an expressed aversion and dislike of members of one’s political outgroup – has increased in many polities in recent years, and thus published research on the topic has proliferated. Studies have asserted that affective polarization is tied to prejudice and authoritarianism, among other potentially harmful phenomena, and is buffered by intellectual humility. We assert that this literature is hindered by the use of *ad hoc*, heterogeneous measures of affective polarization which have not been properly psychometrically evaluated, and which limit research clarity and make cumulative science on the topic difficult. Informed by the common extant measures of affective polarization we constructed a new scale and investigated its reliability and construct validity. In Study 1 we generated items and had them rated by subject matter experts for content validity (*N* = 6). In Study 2, a sample of US participants completed the scale (*N* = 326), an EFA suggested a three-factor model, which had good reliability. In Study 3, a CFA (*N* = 331) confirmed that a three-factor model fit the data, with subscales labelled Social Distance, Aversion, and Incivility. We also showed that our Affective Polarization Scale had good reliability, through the results of the α- and ω-indicators of reliability. Construct validity analyses supported all pre-registered hypotheses, showing that scores on our scale were positively correlated with authoritarianism, need for closure, and identity strength, and negatively correlated with intellectual humility. We make suggestions for future research and scale usage, such as investigating measurement invariance in different populations, or with different outgroup targets.

## Introduction

Social and political discourse globally, in the West specifically, seems to have become increasingly characterised by vitriol, distrust, and resentment between ideological groups in recent years. This apparent polarization has been the subject of a lot of scholarly attention, particularly in Western nations. Initially, polarization was largely defined in terms of ideological polarization, which is the divergence of partisan groups in terms of their positions on major issues (Fiorina et al., 2008). The extent of ideological polarization is debated, however, with some claiming that ideological polarization has increased, exemplified by the apparent loss of moderates in the US; while others claim that most citizens in fact remain centrists (Abramowitz & Saunders, 2008; Fiorina et al., 2008; Fiorina & Abrams, 2008). Many researchers now believe that ideological polarization does not capture the true extent of polarization (Iyengar et al., 2019). Instead, polarization is better described in terms of *affective* polarization, which refers to the level of antipathy felt towards outgroup members (Hetherington et al., 2016; Iyengar et al., 2012; 2019). Even when different ideological groups become more moderate or remain stable in terms of their ideological positions, they can be increasingly affectively polarized, displaying antipathy through avoidance, distrust, schadenfreude (taking pleasure in others’ misfortune), and disliking across groups (Levendusky & Malhotra, 2016; Mason, 2013; 2015; Webster et al., 2024). Affective polarization has increased in the American socio-political environment especially but is also occurring in much of Europe (Boxell et al., 2022; Kekkonen & Ylä-Anttila, 2021; Orriols & León, 2020; Reiljan, 2020; Wagner, 2024).

Most explanations of affective polarization are informed by the social identity approach, and related theories such as intergroup emotions theory (Druckman & Levendusky, 2019; Greene, 1999; Iyengar et al., 2019; Kalin & Sambanis, 2018). These assert that partisanship and political orientation functions as a social identity, which provides a range of benefits to the individual, such as fulfilling epistemic needs, providing a sense of belonging and a sense of collective self-esteem, as well as a cognitive framework for interpreting social phenomena and directing behaviour.

Intergroup emotions theory (Mackie et al., 2008; Smith & Mackie, 2015) focusses on the way emotions are experienced on behalf of one’s social identity group when that group identity is salient, explaining that, in such a context, emotions which arise are often based on group-level rather than individual-level appraisals. Individuals make appraisals of others in their social environment, their group membership, their traits, and their behaviour (Fischer & Manstead, 2008; Smith & Mackie, 2015). Upon appraising an outgroup and its members negatively, as hostile or threatening in some way, emotions arise which perform a social distancing function (Fischer & Manstead, 2008). These social distancing emotions, anxiety and anger, are motivational states which may result in confrontational or avoidant behaviours, depending on the context (Petersen, 2010). Often no simple unidimensional emotional state is experienced, but rather a combination of anger and anxiety which, coupled with contextual factors, produces more specific feelings and states, such as avoidance, schadenfreude, and callousness (Smith & Mackie, 2015).

Affective polarization may cause harm to individuals, and society in general. It has been shown to spill over and influence non-political judgements: people display favouritism towards co-partisans while displaying prejudice towards outgroup members, which in some studies has been seen to exceed discrimination based on race, religion, and gender (Carmines & Nassar, 2022; Iyengar & Westwood, 2015; Westwood et al., 2018). This discrimination has also been observed in the evaluation of job applicants and dating behaviour (Gift & Gift, 2015; Huber & Malhotra, 2017). Some research construes affective polarization in terms of partisans’ desire for “social distance” from their political/ideological outgroups, with partisans displaying a moderate reluctance to live near, be friends with, or have family ties to opposing partisans (Iyengar et al., 2012; Levendusky & Malhotra, 2016). Characterised by distrust and antipathy between groups, affective polarization may lower social trust and undermine healthy social and political processes (Druckman et al., 2013, 2021; Gidron et al., 2019; Kingzette et al., 2021; Torcal & Thomson, 2023). Affective polarization is also associated with cognitive biases which leave one susceptible to misinformation (Jenke, 2023). It likewise contributes to selective exposure and thus echo-chamber formation, or cyberbalkanization, which contributes to the proliferation of mis- and dis-information (Arora et al., 2022; Chan et al., 2019; Lorenzano et al., 2018; Wang & Song, 2020). Those who are more affectively polarized on both the political left and the right show a greater tendency toward authoritarianism (Costello et al., 2022; Johnston, 2018; Luttig, 2017; Renström et al., 2022).

It is therefore important for psychologists to continue to study the antecedents and effects of affective polarization, as it is likely harmful to individuals and society. However, to study affective polarization effectively, there needs to be a clarification and refinement of the measures used. Throughout the literature there is a marked heterogeneity of measures, and there has been little justification or discussion of these various measures (Kubin & von Sikorski, 2021). In this study, we perform an informal review of the measures used to study affective polarization, and strive to develop a new, valid measure.

### Measures of affective polarization

The way affective polarization has been measured in previous studies is incredibly inconsistent, and there is a need for a standard measure with a clear construct definition (Kubin & von Sikorski, 2021). In contrast to ideological polarization, which is the divergence of policy positions or ideological positions between partisans, affective polarization is a construct which concerns *social-emotional* polarization specifically, representing a breakdown of cohesion and good-will between group members. In much of the research on polarization, the term “political polarization” has been used to refer to both ideological and affective polarization, with two-thirds of studies failing to define the construct, and many not explicitly specifying which form of polarization is being studied (Kubin & von Sikorski, 2021). Additionally, the terms behavioural polarization, social polarization, partisan prejudice, and affective polarization have all been used to refer to generally the same construct, which is an expressed dislike and aversion to outgroup members (Garrett & Bankert, 2018; Kubin & von Sikorski, 2021; 2023).

There are three types of measures most commonly used throughout the affective polarization literature: feeling thermometers, trait ratings, and social distance items (Druckman & Levendusky, 2019; Lelkes, 2016). Feeling thermometers ask participants to rate their feelings towards a relevant outgroup on a warmth-coldness scale, usually on a scale of 0 to 100. Trait ratings ask participants to rate a group on a range of traits such as honesty, patriotism, intelligence, selfishness, meanness. Social distance items ask participants how comfortable they would be if their child married a member of a relevant outgroup, or their willingness to befriend outgroup members. Less often, ratings of trust in outgroup members have also been used (Rudolph & Hetherington, 2021). In a study on the moral roots of affective polarization, Garrett and Bankert (2018) used new hostility items pertaining to schadenfreude, anger, antagonism, and incivility. In many studies, multiple measures are used in tandem, and analysed as separate measures of affective polarization (e.g., Iyengar et al., 2012). Sometimes they are combined to form an overall measure of affective polarization (e.g., Huddy & Yair, 2021).

Evidence does suggest that the disparate methods for measuring affective polarization are related. In a review of the measurement of affective polarization, Druckman and Levendusky (2019) found that the feeling thermometer, trust ratings, and trait ratings were all correlated with one another (*r* = 0.44–0.63). Social distance items were not highly correlated with the others in their study, though a study by Renström et al. (2021), which used multiple measures of affective polarization, found no difference in results based on which measure was used in analyses, indicating that they were all highly correlated. Similarly, Costello et al. (2022) and Gidron et al. (2022) found that the social distance items and feeling thermometers were highly correlated, and Huddy and Yair (2021) found that trait ratings, social distance items, and feeling thermometers were all associated with one another. Druckman and Levendusky (2019) used the trait ratings and social distance items as separate scales, both of which showed good reliability (trait ratings, α = .90; social distance items, α = .80). Garret and Bankert (2018) combined the social distance items and hostility items into a scale (α = .87). Huddy and Yair (2021) combined all items (feeling thermometer, trait ratings, and social distance items) to calculate single affective polarization score. This was done for participant ratings of both their in-party (α = .59) and out-party (α = .76). Druckman et al. (2021) combined feeling thermometers, trust ratings, trait ratings, and social distance measures into a single scale (α = .88). However, despite these papers reporting high reliabilities, there has been little analysis of content validity, item wording, or construct validity, and the items used varied widely between studies.

While the feeling thermometer is the original and most widely used measure of affective polarization because of its suitability for large scale political polling, the format is abstract and vague, and has been criticized for poor validity (Lelkes, 2016; Wilcox et al., 1989). In order to accurately capture variation in the construct, it may not be appropriate to simply ask participants directly how warm or cold they feel towards the outgroup. Doing so is vague and often it is difficult for participants to properly summarise their feelings on a single item which is devoid of content reflecting the social context of affective polarization.

We assert that the domains most relevant to affective polarization have therefore been previously identified in the affective polarization literature; the trait ratings are the appraisals upon which the intergroup emotions are based, and the social distance, incivility, and schadenfreude type items reflect the negative and social distancing emotions which arise. However, as described above, these types of items were rarely combined into a content valid scale which reflected the full dimensionality of the construct, and proper analyses of the psychometric properties of the scales were not performed. Rather, the scales were made “on the fly”, resulting in multiple different measures of the same construct, and the respective authors used the common yet problematic practice of simply reporting Cronbach’s alpha as the extent of their psychometric analyses (Flake et al., 2017).

A standard, content-valid, comprehensive affective polarization scale will provide affective polarization research with greater validity, allow for the comparison of results and effect sizes, and make meta-analytic studies easier (Kubin & von Sikorski, 2023). A measure of affective polarization with more items than the simple feeling thermometer or the short scales used previously will likely provide a scale with better reliability, as reliability generally increases with item number, at least for short scales (Spearman, 1910). Additionally, while there may be some situations where single-item feeling thermometers are appropriate, these are not ideal for complex or abstract constructs; multiple items are needed to accurately model measurement error, especially for analyses such as structural equation modelling (Fuchs & Diamantopoulos, 2009; Petrescu, 2013). One also needs to properly investigate content validity, dimensionality, and construct validity, which has not been done for the affective polarization scales. Thus, the present study will investigate these issues, and provide evidence for the validity of a new measure of affective polarization adapted from those used previously.

### Overview

The present studies were informed by Boateng et al.’s (2018) best practices guidelines for developing and validating scales for health, social, and behavioural research. Study 1 began by generating a pool of items and submitting them to six subject matter experts (SMEs) for analysis of content validity. Based on the SME ratings of item appropriateness, we produced item and scale content validity index (CVI) scores.

Study 2 entailed administering the new affective polarization scale (APS) to a sample of participants and performing exploratory factor analyses (EFA) and reliability analysis, to inform the subsequent confirmatory and validity analyses.

In Study 3 we performed fully pre-registered confirmatory tests of dimensionality, reliability, and construct validity. Construct validity was assessed using six related variables from affective polarization’s nomological network: feeling thermometer scores, need for closure, intellectual humility, authoritarianism, and identity strength.

## Study 1: Item Development

### Domain Identification

Based on the research on affective polarization (Iyengar et al., 2019), social identity (Greene, 1999), and intergroup emotions (Mackie et al., 2008; Smith & Mackie, 2015), we arrived at a simple, broad definition: Affective polarization is the degree of antipathy one holds towards their outgroup, expressed as negative appraisals of the outgroup, a desire for social distance from the outgroup, and incivility towards the outgroup.

Based on our definition and theory generated from previous studies, we settled on content sub-domains *a priori*. These domains were to be investigated as actual statistical dimensions of our measure in Study 2, but in this stage they simply informed our attempt to ensure content validity. The domains were:

1. Social Distance: A reluctance to interact with members of the target outgroup, a desire for social distance.
2. Aversion: Trait ratings and negative appraisals of the outgroup.
3. Incivility: Confrontational or callous attitudes and behaviours towards outgroup members, though of a non-violent nature, such as schadenfreude and incivility.

### Item Generation

We used the deductive method, or ‘classification from above’ (Boateng et al., 2018: 5), to generate items based on the descriptions of the domains and assessment of previous scales used throughout the literature. We intended to construct a relatively short scale for researcher and participant convenience. We targeted a scale of approximately 15 items, and therefore generated an item pool at least twice as large (Boateng et al., 2018). We generated 35 items for our initial item pool. Many of these were informed by and adapted from previous measures of affective polarization reviewed above. The blank space in each item represents the target which respondents will be rating (e.g., “Liberals”, “Conservatives”, “Democrats”, “Republicans”, etc.). Items were to be measured on a 7-point Likert scale, which is recommended for bipolar items (Boateng et al., 2018). Response items ranged from *strongly disagree* to *strongly agree* with a neutral point labelled *neither*.

### Content Validity

The construct definition, the content domains, and the initial 35 items were presented to a panel of six psychologists who were asked to rate item appropriateness on a 4-point Likert scale, for calculation of the content validity index (CVI, Yusoff, 2019). All subject matter experts had a PhD in psychology, and currently work as researchers and educators in the field of psychology. Table 1 shows the initial 35 items along with the item CVI score, which is the proportion of experts in agreement as to item relevance to its corresponding domain.

**Table 1:** Initial items, item CVI scores, and scale CVI score.


DOMAIN	ITEM	EXPERTS IN AGREEMENT	I-CVI

Social Distance	I would be upset if my friend married a ____.	5	0.8

	I would be upset if a close family member married a ____.	5	0.8

	I would not like it if a ____ moved into the house next door.	5	0.8

	I avoid forming friendships with ____.	6	1

	I would not go into business with a ____.	5	0.8

	I try to avoid socialising with ____.	6	1

	I would not be willing to date a ____.	5	0.8

	I block or unfriend ____ on social media.	4	0.7

	I do not follow ____ on social media.	5	0.8

	I avoid ____ areas.	4	0.7

Aversion	____ are untrustworthy.	6	1

	I dislike ____.	6	1

	____ are dishonest.	4	0.7

	____ are selfish.	3	0.5

	____ are hypocritical.	5	0.8

	____ are mean.	4	0.7

	____ are immoral.	5	0.8

	____ are unfriendly.	6	1

	____ are judgemental.	3	0.5

	____ are cold.	4	0.7

	____ are dangerous.	6	1

	____ are rude.	4	0.7

Incivility	I do not help ____ when they need it.	4	0.7

	I am happy when negative things happen to ____.	6	1

	I would be happy if there were no ____.	5	0.8

	I try not to support ____ businesses.	5	0.8

	I like to see ____ taken down a peg.	5	0.8

	____ need to be punished for the harm they cause.	5	0.8

	When bad things happen to ____ it is their own fault.	5	0.8

	____ do not deserve sympathy.	5	0.8

	I am happy when ____ look bad.	5	0.8

	I like to cause ____ trouble.	4	0.7

	I like to make ____ angry.	4	0.7

	I like to see ____ put in their place.	5	0.8

	It is appropriate to mock ____.	5	0.8


Based on the recommendations of Polit et al. (2007), when using six subject matter experts, items with five or more experts in agreement of item relevance (an I-CVI of 0.8) were retained in the scale for further investigation. Based on this, 24 items would have been retained. However, we also chose to retain the items ‘I like to make ____ angry’, and ‘I block or unfriend ____ on social media’, as variations of these were used in a reliable measure of affective polarization previously (Garrett & Bankert, 2018), and we believe they are likely relevant to the hostility and social distance domains respectively. Likewise, variations of the item ‘____ are mean’ were used previously in studies (Druckman & Levendusky, 2019) so we retained it at this stage also. These items had an I-CVI of 0.67, which is judged by Polit et al. (2007) as *fair*. This resulted in a scale of 27 items to be subjected to EFA.

## Study 2: EFA and Reliability

This study aimed to explore structure of the Affective Polarization Scale and also to examine the preliminary evidence for its reliability.

## Method

### Participants

A total of 326 participants were recruited from Prolific. Participants were required to be American residents to be eligible to participate. The average age of participants was 36 (SD = 12.4), with three participants not stating their age. Sex was 50% male/female. The sample was approximately 80% white, 6% Asian, 5% black, 7% mixed, and 2% other. Of these, 143 (44%) described themselves as ‘liberal’, 74 (23%) as ‘slightly liberal’, 46 (14%) as ‘neither’, 38 (12%) as ‘slightly conservative’, and 25 (7%) as ‘conservative’. Of the 46 who identified as neither liberal nor conservative, 16 stated that they least identify with liberals and thus rated a liberal target for the affective polarization scale, while 30 stated that they least identify with conservatives, these participants rated a conservative target for the affective polarization scale. There was no missing data.

### Measures

Participants were asked about their political ideology with possible response items: *Liberal, Slightly liberal, Neither, Slightly conservative, Conservative*. Those who responded *Neither* were asked to *Please indicate which group you least identify with*, with options *Liberal* or *Conservative*.

Though there may be some issues with using simplified categories of political ideology (Costello et al., 2023), there is also evidence that such simplified political labels do reflect the manner in which polarization drives people to sort into overarching ‘blocs’ (Bantel, 2023; Comellas & Torcal, 2023; Kekkonen & Ylä-Anttila, 2021; Knudsen, 2021; Orriols & León, 2020; Oshri et al., 2022; Somer & McCoy, 2018; Wagner, 2021). Likewise, while some people are largely distrustful of politicians and parties, most nevertheless align with and hold sets of beliefs which are reflective of the broad worldviews underlying those political divisions or affective blocs (Haidt, 2012; Pinker, 2002; Sowell, 2007). We opted for labels which refer to the broad Liberal versus Conservative ideological divide rather than to party labels like Democrat and Republican, as these party labels may conjure images of politicians or party elites in participants’ minds, and people’s feelings towards party elites versus everyday party aligners is often quite different (Druckman & Levendusky, 2019).

The affective polarization scale consisted of 27 Likert type items with responses ranging from *Strongly disagree* to *Strongly agree*. Nine of these items were inspired by the social distance items used in previous studies, seven items were trait ratings, and 11 items pertained to schadenfreude and incivility.

### Procedure

Participants who were eligible for the present study were invited to participate. Those who chose to participate were given a brief description of the study, before being asked about their political ideology. Those who selected liberal, or who identified least with conservatives, were assigned an embedded tag of “Conservative”, which the software used as the target for their survey questions. Those who were conservative or who least identified with liberals were tagged “Liberal”, this was inserted into the questions for these participants. Each participant was therefore rating their level of affective polarization from their outgroup. Participants were instructed to indicate their level of agreement with the 27 items, after which they were given a short debrief and redirected to Prolific. Low-risk ethics approval was obtained from the first author’s institution, and participants were adequately remunerated for their participation according to Prolific’s payment guidelines.

## Results

We ran an exploratory factor analysis on all 27 items, with Principal Axis Factoring and an Oblimin rotation. We used a parallel analysis to decide which factors to retain, because of the known strength of this method (Horn, 1965; Velicer & Jackson, 1990). This resulted in a three-factor solution which cumulatively explained 67.1% of the common variance. With a few exceptions, the factor analysis retained the three domains of content as separate coherent factors (Table 2).

**Table 2:** Initial factor loadings for the full 27 item affective polarization scale.


	FACTOR		

	1	2	3

I would be upset if my friend married a ____.	0.93		

I would be upset if a close family member married a ____.	0.79		

I would not like it if a ____ moved into the house next door.	0.79		

I avoid forming friendships with ____.	0.84		

I would not go into business with a ____.	0.63		

I try to avoid socialising with ____.	0.76		

I would not be willing to date ____.	0.47		0.41

I block or unfriend ____ on social media.			0.35

I do not follow ____ on social media.	0.49		

____ are untrustworthy.			0.59

I dislike ____.			0.47

____ are hypocritical.			0.79

____ are immoral.			0.7

____ are unfriendly.			0.61

____ are dangerous.			0.62

____ are mean.			0.83

I am happy when negative things happen to ____.		0.92	

I would be happy if there were no ____.		0.39	

I try not to support ____ businesses.	0.44		

I like to see ____ taken down a peg.		0.68	

____ need to be punished for the harm they cause.		0.60	

When bad things happen to ____ it is their own fault.		0.63	

____ do not deserve sympathy.		0.66	

I am happy when ____ look bad.		0.71	

I like to make ____ angry.		0.77	

I like to see ____ put in their place.		0.69	

It is appropriate to mock ____.		0.69	

Variance explained (%)	22.9	23.5	20.7


*Note*: Loadings below 0.3 were suppressed.

In order to shorten the scale to a convenient length, and to remove items with low loadings, we selected the five highest loading items from each domain and repeated the analysis. The parallel analysis again suggested a three-factor solution, which cumulatively explained 72% of the common variance (Table 3).

**Table 3:** Factor loadings of the 15 highest loading items from the full scale.


	FACTOR		

	1	2	3

I would be upset if my friend married a ____.	0.99		

I would be upset if a close family member married a ____.	0.86		

I would not like it if a ____ moved into the house next door.	0.75		

I avoid forming friendships with ____.	0.71		

I try to avoid socialising with ____.	0.64		

____ are hypocritical.		0.79	

____ are immoral.		0.73	

____ are unfriendly.		0.70	

____ are dangerous.		0.68	

____ are mean.		0.93	

I am happy when negative things happen to ____.			0.88

I am happy when ____ look bad.			0.75

I like to make ____ angry.			0.74

I like to see ____ put in their place.			0.66

It is appropriate to mock ____.			0.70

Variance explained (%)	25.1	25.3	21.3


*Note*: Loadings below 0.3 were suppressed.

We calculated subscale average scores as well as an overall average score from the 15 items. The correlations between the subscale averages and the scale average were analysed using Pearson’s correlations (Table 4). While the subscale average scores were quite strongly correlated with one another, the overall scale average was very strongly correlated with each subscale average.

**Table 4:** Correlations between subscale average scores and overall scale average scores.


	SCALE AVERAGE	SD AVERAGE	A AVERAGE	I AVERAGE

Scale Average	—			

SD Average	0.91***	—		

A Average	0.92***	0.77***	—	

I Average	0.87***	0.65***	0.71***	—


*Note*: *** indicates *p* < .001.

Lastly, we calculated reliability coefficients for all three factors and an overall reliability score for a single factor containing all 15 items (Table 5). All reliabilities were very high, indicating that the proportion of the variance in the responses to our scale which is attributable to true construct variance is very high.

**Table 5:** Cronbach’s α coefficients for the three factors and a single factor containing all 15 items.


FACTOR	CRONBACH’S α

Social Distance	.94

Aversion	.93

Incivility	.90

Full scale	.96


*Note*: Macdonald’s *ω_t_* coefficients were also calculated, all were within 0.002 of the corresponding *α*’s.

## Study 3: Confirmatory Factor Analysis and Construct Validity

### Introduction

Study 3 entailed investigating the factorial and construct validity of the affective polarization scale. The analyses for this study were pre-registered (https://osf.io/3srt8/?view_only=3f9953c2c88c4499baac89a26614055c), and the survey materials, de-identified data, and the R-code can be found at: https://osf.io/bgx2r/?view_only=9d0429f3502d49c2940a3913bd7a64e3.

Firstly, a confirmatory factor analysis (CFA) was performed, testing the three-factor model seen in Study 2. We hypothesised (H1) that the three-factor model with correlated factors would show acceptable fit, based on the inference criteria outlined in the pre-registration.

Construct validity was assessed by analysing correlations between affective polarization scores and constructs which have seen to correlate with other measures of affective polarization.

#### Feeling Thermometer

To investigate convergent validity, we assessed the correlation between scores on the feeling thermometer and our affective polarization scale. Druckman and Levendusky (2019) found that the scores on the feeling thermometer were significantly correlated with scores on the trait ratings and trust items. Costello et al. (2022) and Gidron et al. (2022) found that social distance items and the feeling thermometer were highly significantly correlated. Likewise, Huddy and Yair (2021) found that scores on the feeling thermometer were closely related to all other measures of affective polarization.

Therefore, we hypothesised (H2) that the subscale and overall affective polarization scores would be significantly negatively correlated with the feeling thermometer.

#### Authoritarianism

Authoritarianism is a personality construct which reflects a desire to enforce conformity over personal autonomy, to wield and or submit to group authority, and to punish transgressors of the groups’ valued norms. Authoritarianism is most commonly studied in its right-wing form, though there is debate about the nature of authoritarianism and the existence of left-wing authoritarianism (LWA). However, LWA is gaining favour in the research community (Conway et al., 2018, 2021; Costello et al., 2022; Costello & Patrick, 2022; Krispenz & Bertrams, 2023).

Measures of right-wing authoritarianism (RWA) (Costello et al., 2022; Renstrom et al., 2022), LWA (Costello et al., 2022) and also a politically neutral measure of authoritarianism (Johnston, 2018; Luttig, 2017) have all been shown to correlate with affective polarization. Therefore, we hypothesise that LWA will be positively correlated with subscale and overall affective polarization scores among liberal participants (H3a), as will RWA among conservative participants (H3b).

#### Identity Strength

Processes of social identity underpin affective polarization (Greene, 1999; Iyengar et al., 2019). Those who more strongly identify with their political/ideological group, those for whom this identity is more central, show higher levels of affective polarization (Huddy et al., 2015; Iyengar & Westwood, 2015; Luttig, 2018; Westwood et al., 2018). For this reason, we hypothesise that identity strength will be positively associated with subscale and overall affective polarization scores (H4).

#### Intellectual Humility

Bowes et al. (2020) found that both politics-specific and general intellectual humility was negatively correlated with affective polarization across multiple measures of both constructs, and in multiple studies. Krumrei-Mancuso and Newman (2020) found similar results, with those higher in intellectual humility showing less ingroup favouritism on feeling thermometer ratings. Similarly, Nadelhoffer et al. (2020) found that measures of political animosity and feeling thermometer scores were negatively correlated with intellectual humility. Stanley et al. (2020) also found that those who are low in intellectual humility were less willing to befriend opponents and were more derogatory of the opponents’ moral and intellectual character. Therefore, we hypothesise that intellectual humility will be negatively correlated with subscale and overall affective polarization scores (H5).

#### Need for Closure

Need for closure (NFC) is the degree to which a person feels averse to uncertainty, ambiguity, and confusion. Kruglanski and Webster (1996) describe it as a desire for definite knowledge and a motivated closing of the mind. It describes a style of motivated cognition in which individuals tend to ‘seize’ and then ‘freeze’ on an answer or idea in order to settle ambiguity. Need for closure (both self-report scales and behavioural measures of cognitive flexibility) is thought to be a significant component driving a range of group-based phenomena such as polarization, prejudice, and authoritarianism, as the strong need for closure results in prejudice and aversion to groups which present a threat to certainty and closure (Costello et al., 2022; Dhont et al., 2011; Luttig, 2018; Roets & Van Hiel, 2006; Zmigrod et al., 2020). Therefore, we hypothesize that NFC will be positively correlated with subscale and overall affective polarization scores (H6).

## Method

### Participants

We performed a power analysis for all of the pre-registered analyses. The power analysis for the CFA was performed using the *pwrSEM* web application (https://yilinandrewang.shinyapps.io/pwrSEM/). We estimated power using both the ‘power analysis for parameter estimation’ and the ‘power to detect model misspecification’ methods. Based on the three-factor model from Study 2 and the loadings seen therein, we calculated that a sample size of 300 participants would have adequate power.

For the correlational analyses, we performed a power analysis in G*Power. Based on a power of 80% to detect an effect size of *r* = .2, a sample of at least 193 was required. The power analyses can be seen in more detail in the pre-registration (https://osf.io/3srt8/?view_only=3f9953c2c88c4499baac89a26614055c).

Three hundred and forty-one American Prolific users were recruited for the study. Given the somewhat unbalanced numbers of liberal versus conservative participants in Study 1, we pre-screened for participants who self-described as liberal or conservative in their Prolific profile and recruited 170 of each. As per the exclusion criteria laid out in the pre-registration, we excluded data from 10 participants. Seven did not pass the attention check and three failed to answer the question confirming that their data was valid. This left a final sample of 331 participants, with a mean age of 41.5 years (SD = 14.4); gender and political ideology characteristics of the participants can be seen in Table 6.

**Table 6:** Descriptive statistics of participant gender and political ideology.


CHARACTERISTICS	*n*

Gender	

Female	153 (46.4%)

Male	167 (50.6%)

Non-binary	9 (2.7%)

Prefer not to say/did not respond	2 (0.6%)

Political ideology	

Liberal	114 (34.4%)

Slightly Liberal	53 (16%)

Neither	4 (1.2%)

Slightly conservative	82 (24.8%)

Conservative	78 (23.6%)


Of the four participants who indicated they were neither liberal nor conservative, three indicated that they identify least with conservatives, and thus rated the Conservative target for the affective polarization scale and completed the left-wing authoritarianism scale. The single participant who indicated they least identify with liberals completed the right-wing authoritarianism scale and rated a Liberal target for the affective polarization scale.

### Measures

#### Feeling Thermometer

Participants rated their feelings of warmth towards their outgroup (Liberals or Conservatives) on a 101-point scale, from *Cold (0)* to *Warm (100)*.

#### RWA

We measured RWA using Altemeyer’s (1996) 22-item RWA scale (α = .92). It taps three dimensions: authoritarian aggression, conventionalism, and authoritarian submission. It contains items such as ‘Our country will be destroyed someday if we do not smash the perversions eating away at our moral fibre and traditional beliefs’ and was measured on a 7-point scale from *strongly disagree* to *strongly agree* (unless otherwise indicated, all of the following scales were measured on the same 7-point scale).

#### LWA

We measured LWA using the LWA-25 scale (α = .91). It contains items such as ‘We need to replace the established order by any means necessary’. It taps three dimensions: anti-hierarchical aggression, anti-conventionalism, and top-down censorship (Costello & Patrick, 2022).

#### Intellectual Humility

We measured intellectual humility the nine-item specific intellectual humility scale (Hoyle et al., 2016). This scale (α = .92) is unidimensional and measures intellectual humility in a specific domain using items such as ‘I am open to new information in the area of Politics that might change my view’.

#### Need for Closure

We measured need for closure using the 15-item version (α = .91) of the Need for Closure Scale (Roets & Van Hiel, 2011). It is a unidimensional scale and contains items such as ‘I dislike questions which could be answered in many different ways’.

#### Identity Strength

Identity strength was measured using the four-item scale (α = .88) from Huddy et al. (2015). Items include ‘When talking about [Liberals/Conservatives], how often do you use “we” instead of “they”?’. Responses were collected on a 5-point scale from *not at all* to *a great deal*.

#### Affective Polarization

Affective polarization was measured using the 15-item affective polarization scale developed in the present study (Table 3). This scale measured affective polarization in terms of social distance, aversion, and incivility (α = .96).

### Procedure

Participants began the survey by consenting to participate and then answering the demographic and political ideology questions. Those who indicated that they were liberal or slightly liberal were given an embedded tag of ‘Target = Conservative’ and ‘Group = Liberal’. The target label was automatically inserted into the items for the affective polarization scale, and the survey was programmed to show participants in the liberal group the left-wing authoritarianism scale. Those who indicated they were conservative or slightly conservative received embedded tags ‘Target = Liberal’ and ‘Group = Conservative’. Thus, they rated a liberal target for the affective polarization scale and completed the right-wing measure of authoritarianism. The Group tag was also automatically inserted into the identity strength items, such that participants were rating their feelings of identification towards their own ideological label.

After completing the demographic questions, participants were randomly presented with the affective polarization, RWA/LWA, identity strength, specific intellectual humility, feeling thermometer, and need for closure scales, as well as an attention check. The attention check was an instructional attention check: ‘The mood test you are about to take part in is very simple, when asked how you are feeling you must select “Neutral”. This is an attention check.’ Participants who did not select the indicated option were considered to have failed the check. Items within all scales were randomized. Participants then answered questions pertaining to conspiracy theory beliefs, for a related project. The final question asked participants if there is any reason that we should not use their data, participants were assured of payment regardless of their response. Low-risk ethics approval was obtained from the first author’s institution, and participants were adequately remunerated for their participation according to Prolific’s payment guidelines.

### Statistical Analyses

All analyses were performed in R version 4.2.2. Our hypotheses and inference criteria were fully pre-registered: https://osf.io/3srt8/?view_only=3f9953c2c88c4499baac89a26614055c. We used the *lavaan* package (v0.6.17; Rosseel, 2012) to perform the CFA. The model tested in the CFA was that from the EFA in study 2, with the five social distance items, five aversion items, and five incivility items loading on separate correlated factors. Because of the ordinal nature of the indicator items, we used the ‘ordered = TRUE’ argument in *lavaan*, which uses the DWLS estimator to estimate model parameters, and the WLSMV to estimate robust standard errors and test statistics (The Lavaan Project, n.d.). Pearson’s correlations between the affective polarization scale and the construct validity scales were analysed.

## Results

### Hypothesis 1

**Figure 1:**
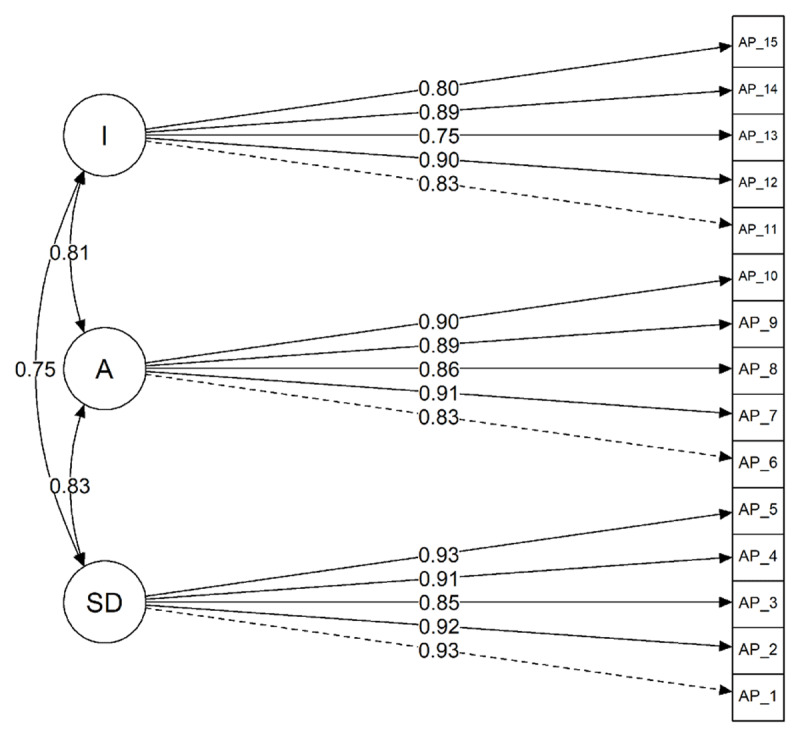
A path model showing the factor structure of the affective polarization scale, using standardized coefficients. *Note:* Item numbering corresponds to item order in Table 3. SD = Social Distance, A = Aversion, I = Incivility.

We assessed fit of the three-factor model using the χ^2^ test statistic, RMSEA, SRMR, TLI, and CFI statistics. The chi-square statistic did not indicate perfect fit: χ^2^(87) = 397.39, *p* < .001. Likewise, the RMSEA statistic was beyond the <.08 cut-off (RMSEA = 0.10 [0.09. 0.11]). In contrast, the SRMR statistic indicated very good fit (0.03), as did the TLI (0.93), and CFI (0.94). The measurement model, with standardised parameter estimates, can be seen in Figure 1.

As acknowledged in our pre-registration, a significant chi-square test statistic would not be taken as good evidence against the model fit, as the over-sensitivity of this test under larger sample sizes is well known; it rejects models for even slight misfitting especially with non-normal data and highly correlated indicators (Boateng et al., 2018). We also acknowledged that when assessing fit using a variety of fit statistics, there is likely to be some level of disagreement between them which necessarily results in a certain amount of researcher degrees of freedom in interpretation of those statistics.

Given that three of the four main measures of fit indicated very good fit, we take this as evidence that the three-factor model adequately fits the data. Hypothesis 1 is therefore considered to be supported.

### Reliability

While we did not preregister our reliability analyses, we calculated reliabilities for all the scales used in the present study (Table 7). The affective polarization scale had good alpha reliability (α), as did the rest of the scales used. Total omega (ω_t_) calculates the total reliability of a scale, while loosening unrealistic assumptions associated with α, such as that of tau-equivalence and uni-dimensionality (Flora, 2020). The fact that the affective polarization scale has good ω_t_ reliability indicates that a high proportion of variance in both total and subscale scores is attributable to true score, and this means the scale can likely be used to calculate total and subscale scores. All construct validity scales likewise had good reliability.

**Table 7:** Cronbach’s alpha and McDonalds omega total for the scales used.


SCALE	Α	ω_t_

Affective Polarization	.96	.97

Need for Closure	.91	.93

Identity Strength	.88	.89

Specific Intellectual Humility	.92	.94

RWA	.92	.94

LWA	.91	.93


### Hypothesis 2

Hypothesis 2 was supported. All three affective polarization subscales were negatively correlated with the feeling thermometer (Social Distance: *r*(329) = *–*.54, *p* < .001; Aversion: *r*(329) = –.63, *p* < .001; Incivility: *r*(329) = –.47, *p* < .001), as was the overall scale score (*r*(329) = –.61, *p* < .001).

### Hypothesis 3a

Hypothesis 3a was supported. Among Liberal participants, all three affective polarization subscales were positively correlated with LWA (Social Distance: *r*(168) = .62, *p* < .001; Aversion: *r*(168) = .62, *p* < .001; Incivility: *r*(168) = .57, *p* < .001), as was the overall scale score (*r*(168) = .67, *p* < .001).

### Hypothesis 3b

Hypothesis 3b was supported. Among Conservative participants, all three affective polarization subscales were positively correlated with RWA (Social Distance: *r*(159) = .44, *p* =< .001; Aversion: *r*(159) = .42, *p* =< .001; Incivility: *r*(159) = .25, *p* = .001), as was the overall scale score (*r*(159) = .41, *p* =< .001).

### Hypothesis 4

Hypothesis 4 was supported. All three affective polarization subscales were positively correlated with Identity strength (Social Distance: *r*(329) = .25, *p* < .001; Aversion: *r*(329) = .25, *p* < .001; Incivility: *r*(329) = .19, *p* < .001), as was the overall scale score (*r*(329) = .25, *p* < .001).

### Hypothesis 5

Hypothesis 5 was supported. All three affective polarization subscales were negatively correlated with specific intellectual humility (Social Distance: *r*(329) = –.29, *p* < .001; Aversion: *r*(329) = –.33, *p* < .001; Incivility: *r*(329) = –.22, *p* < .001), as was the overall scale score (*r*(329) = –.31, *p* < .001).

### Hypothesis 6

Hypothesis 6 was supported. All three affective polarization subscales were positively correlated with the need for closure (Social Distance: *r*(329) = .21, *p* < .001; Aversion: *r*(329) = .21, *p* < .001; Incivility: *r*(329) = .16, *p* < .001), as was the overall scale score (*r*(329) = .21, *p* < .001).

## Discussion

The purpose of this study was to create and validate an affective polarization scale informed by the literature on affective polarization and the common items with which it is measured. The affective polarization literature is characterised by a wide array of ad-hoc short scales, and authors often use the same construct name and compare results generated by these heterogeneous scales. We generated items informed by those used in the past and informed by common theories of intergroup behaviour and emotions, assessed them for content validity, and performed reliability and construct validity analyses for the new scale.

The Affective Polarization Scale (APS), developed herein, is composed of three subscales. The Social Distance subscale is composed of items which measure participants’ desire for social distance from the target outgroup, that is, a reluctance to form family ties and friendships, a lack of interest in socialising with the outgroup, and a desire for geographical separation. The Aversion subscale pertains to participants’ appraisals of the outgroup, measured through trait ratings indicating the degree to which one appraises the target outgroup negatively. The Incivility subscale measures callousness, schadenfreude, and incivility towards the outgroup.

The CFA confirmed that this three-factor structure fit the data well, and the measure of omega reliability, which account for dimensionality in the data, indicated very good reliability. The scale will allow researchers to investigate results based on fine-grained subscale scores, while also lending itself to an overall affective polarization score. Previously, most researchers have simply used a small number of items from a single domain of affective polarization content, so using an average score of our three-factor content-valid scale, which has shown high correlations with subscale scores, is likely to be an improvement over previous research.

We also showed that the scale has good construct validity. Scores on our scale were shown to significantly correlate with the original measure of affective polarization, the feeling thermometer. Scores were also significantly positively associated – in support of our hypotheses – with related constructs such as left and right-wing authoritarianism, identity strength, and need for closure. We also replicated here the finding that affective polarization is negatively associated with intellectual humility. These observed correlations are interesting contributions the literature on affective polarization, and this speaks to the utility of the APS, as well as providing evidence of the scale’s construct validity.

Therefore, in constructing and validating this scale we have helped to address the issues pointed out by Kubin and von Sikorski (2021; 2023), in that we have produced a validated scale of affective polarization which can bring regularity to the affective polarization literature and improve the ability of researchers to engage in cumulative science. Adopting the APS will allow for meaningful comparisons of effects and associations across studies, which was previously impeded by the ad-hoc nature of affective polarization measurement.

### Limitations and Future Directions

The APS is a scale that can be adapted to measure affective polarization between a participant and any relevant outgroup which can be inserted into the items. In the present study we used political orientation labels Liberal and Conservative, though previous studies have used a variety of relevant targets in this domain, such as Republican versus Democrat, or labels indicating outgroup elites or civilians specifically (Druckman & Levendusky, 2019). It may be that the factor structure of the scale varies depending on target appraised and sample population. Therefore, each time the scale is used – and especially when a new target is being used or the scale is being used in a new context or population – performing a CFA would be recommended, especially if the intention is to use subfactor scores. The R code published alongside this study provides researchers with an easy method for performing these analyses. Additionally, testing measurement invariance in sample sub-groups is also recommended. Because of funding and sample size limitations, we did not test measurement invariance of the scale in Liberals and Conservatives separately, and this is something that may need to be investigated in future studies.

Investigation of “norms” for scores on our scale might also be useful. Given that a certain level of ingroup-outgroup favouritism appears to be quite automatic, it is not clear yet what level of affective polarization should be considered normal or benign. It may be that people do express affective polarization to a certain degree, but whether this translates into intentional real-world prejudice is unclear (Lelkes & Westwood, 2017). Therefore, investigating the relationship between scores on our scale and real-world outcomes, rather than correlations with theoretically related psychometric constructs, will be highly informative.
